# Evaluation of conventional and CT‐based radiostereometric analysis for inducible displacement measurements after total hip arthroplasty

**DOI:** 10.1002/jor.25981

**Published:** 2024-10-01

**Authors:** Jennifer S. Polus, Bart L. Kaptein, Edward M. Vasarhelyi, Brent A. Lanting, Matthew G. Teeter

**Affiliations:** ^1^ School of Biomedical Engineering, Faculty of Engineering Western University London Ontario Canada; ^2^ Department of Orthopaedics Leiden University Medical Center Leiden The Netherlands; ^3^ Division of Orthopaedic Surgery, Schulich School of Medicine & Dentistry Western University London Ontario Canada; ^4^ Department of Medical Biophysics, Schulich School of Medicine & Dentistry Western University London Ontario Canada

**Keywords:** aseptic loosening, computed tomography, corail stem, implant migration

## Abstract

Though radiostereometric analysis (RSA) is the gold standard for migration tracking, computed tomography‐based RSA (CT‐RSA) does not require marker beads and is available for clinical adoption. This study investigated CT‐RSA in comparison to RSA for assessing hip implant stability with inducible displacement (ID) examinations. Patients (*n* = 48) from a previous study returned to be re‐examined for femoral stem stability with CT‐RSA and RSA imaging. Implant migration since patients last follow‐up was calculated as a measure of stability. ID was assessed between alternated leg rotation scans for CT‐RSA and between supine and weight‐bearing scans for RSA. Measurements from ID and double examinations were compared between CT‐RSA and RSA. All stems were well‐fixed with migration <0.2 mm/year. ID measurements were lower with CT‐RSA than RSA for distal translation (mean difference = 0.122 mm, *p* < 0.0001), total translation (mean difference = 0.158 mm, *p* < 0.0001), and total rotation (mean difference = 0.449°, *p* < 0.0001). The ID and double exam were significantly different for total translation and total rotation for CT‐RSA, and significantly different for medial, distal, and total translation, and total rotation for RSA. Precision ranged from 0.049 to 0.130 mm in translation and 0.061° to 0.220° in rotation for CT‐RSA, and from 0.108 to 0.269 mm in translation and 0.151° to 0.670° in rotation for RSA. ID measurements from both CT‐RSA and RSA were minimal, consistent for a cohort with well‐fixed stems. CT‐RSA demonstrated superior precision in all axes compared to RSA. Clinical Significance: Future work should explore the use of CT‐RSA in patients with suspected loosening as a potential diagnostic tool.

## 1 INTRODUCTION

With the aging population and its increasing use in younger patients, the number of total hip arthroplasty (THA) procedures performed annually continues to rise.^1^, ^2^ Despite its notable success rate, complications leading to revision surgery still occur and are increasing alongside the rise in primary THA surgeries.^3^ A recent registry study from seven countries found a THA revision rate of 9.3%, with aseptic loosening being the most common cause, accounting for 35.1% of cases.^4^ Aseptic loosening is characterized as the gradual loosening of implant components without the presence of an infection.^5^ Persistent pain often indicates the need for revision surgery, yet the decision to proceed is multifaceted and relies on a combination of patient‐reported symptoms, clinical assessments, and plain radiographs.^6^ Revision surgery for a loose component is often less complex and can relieve patients' pain and restore function. However, revising a well‐fixed component presents a great surgical challenge and heightened risks for the patient.^7^, ^8^ Therefore, ensuring a definitive diagnosis before proceeding with revision surgery for aseptic loosening is critical for optimizing patient outcomes and ensuring necessary and timely intervention.

Despite it being a prevalent cause for revision surgery, aseptic loosening can pose a diagnostic challenge for surgeons, as there is no clinical tool to definitively assess osseointegration and implant fixation in vivo.^6^, ^9^ Radiostereometric analysis (RSA) is a well‐validated gold standard for implant migration tracking and is commonly used in research settings to measure in vivo movement of implants to ensure stability over time. However, the specialized imaging equipment and the need for tantalum beads to be implanted at the time of surgery limit RSA's clinical applicability. Consequently, RSA cannot be used as a routine clinical tool to measure implant stability in patients who are suspected to have aseptic loosening.^10^, ^11^

Recently, the use of computed tomography (CT) to measure implant migration has been described in literature and has shown precision and accuracy in line with RSA.^10^, ^12^, ^13^, ^14^, ^15^ Unlike RSA, CT equipment is widely available, and CT‐RSA does not require any intraoperative marker beads for analysis of implant migration. However, special consideration of radiation dose is important with CT imaging, as the higher radiation dose compared to an RSA exam may limit the clinical use of CT‐RSA. Still, as CT technology continues to improve in terms of image quality and metal artifact reduction techniques, faster processing times, and reduced ionizing radiation dose, the clinical utility of CT‐based RSA (CT‐RSA) presents itself as a viable diagnostic tool for suspected aseptic loosening.^15^ Inducible displacement exams can be leveraged to help diagnose implant loosening clinically by taking images in loaded and unloaded positions during a single visit. This approach can capture in vivo measurements of implant movement occurring instantaneously under the external load, providing an assessment of potential loosening.^16^, ^17^, ^18^

Given the advancements and widespread availability in CT technology, and the growing adoption of CT‐RSA, the purpose of this study was to investigate the use of CT‐RSA for assessment of implant stability in comparison to the gold‐standard RSA. Specifically, this study evaluated inducible displacement of the femoral stem using both CT and RSA techniques in a patient cohort 5‐years post‐THA. We hypothesized that inducible displacement measured with both conventional and CT‐RSA would be low, and that precision would be equivalent between the two techniques. In addition, longitudinal 5‐year migration data of the Corail femoral stem was reported and used as a reliable measure of implant stability in this cohort.

## 2 METHODS

### 2.1 Study design and participants

This is a level 2 prospective cohort study where patients (*n* = 79) who previously participated in a 2‐year prospective RSA study were invited to return at 5‐years postoperation to be re‐examined for implant stability. Results from the previous study have been reported.^19^, ^20^ The present study was approved by our institutional research ethics board and registered with ClinicalTrials.gov (NCT05893563). Willing participants were recruited and provided written informed consent before participation in this clinical validation study. Patients who were undergoing a primary THA for end‐stage hip osteoarthritis were eligible to participate in the original prospective study. Patients were screened and excluded if they had a body mass index greater than 40 kg/m², symptomatic contralateral osteoarthritis, bilateral or revision THA procedures, cognitive defects or neuromuscular disorders that would prevent a walking test, inability to understand English, and if they lived more than 100 km from our institution. Patients underwent either the direct lateral or the direct anterior approach depending on their surgeon referral and were randomized to receive either a collared or collarless Corail cementless femoral stem (DePuy Synthes). As required for each patient, the surgeon used a standard or a high‐offset stem, along with either a 28, 32, or 36 mm cobalt–chromium femoral head. All patients received a cementless Pinnacle acetabular cup (DePuy Synthes), with an AltrX highly crosslinked acetabular liner (DePuy Synthes) as the acetabular component. A minimum of six 1 mm tantalum beads were inserted into the cortical bone of the proximal femur intraoperatively to enable RSA implant migration tracking. Patient demographic and surgical information was collected from the hospital database and reported (Table 1).

**Table 1:** Demographics and surgical details of the patient cohort.

Characteristic	Patients cohort details (*n* = 48)
Sex	25 (52%) Men, 23 (48%) Women
Age (years)	71.4 (50–90)
Body mass index (BMI) (kg/m^2^)	28.0 (19.8–38.0)
Time since surgery (years)	5.12 (4.00–6.00)
Surgical approach	14 (29%) direct lateral approach 34 (71%) direct anterior approach
Femoral stem type	27 (56%) collarless 21 (44%) collared

*Note*: Values are the actual numbers with percentage or reported as mean with ranges.

### 2.2 Conventional RSA analysis

Patients underwent RSA imaging in both supine and standing positions, with a double examination in the supine position. Patients were instructed to be weight‐bearing on their index leg for the standing exam. A uniplanar calibration cage (RSA Biomedical) was used to define the coordinate system, with positive translation directions defined as proximal translation in the y‐axis, medial translation in the x‐axis, and anterior translation in the z‐axis. Positive rotation directions were defined as internal rotation about the y‐axis, anterior tilt about the x‐axis and valgus rotation about the z‐axis. Model‐based RSA (MBRSA) software (RSAcore) was used to analyze the acquired images. Briefly, MBRSA software aligns virtual projections from computer‐aided design (CAD) models of implants to the actual implant contours on the x‐ray images, accurately defining the implant's spatial position and orientation.^21^ Condition number and mean rigid body error from MBRSA were assessed for femoral bone marker distribution and stability. Condition numbers ranged from 17.6 to 84.9 and mean ± standard deviation of the mean rigid body error was 0.167 mm ± 0.076 mm, below the recommended thresholds of 120 and 0.35 mm respectively.^22^, ^23^ Implant movement since the patient's baseline day‐of‐surgery supine exam in the previous study to this 5‐year postoperation supine exam was measured. Implant stability at 5‐years postoperation was described as average implant subsidence along the y‐axis between the patient's 2‐year and 5‐year exams. Inducible displacement measurements from RSA were calculated in MBRSA as the movement of the femoral stem between the supine and standing exams at the 5‐year follow up visit.

### 2.3 CT‐RSA analysis

Patients underwent CT imaging (Aquilion ONE, Canon Medical Systems Corporation) while supine, with their leg both fully externally rotated (reference scan) and fully internally rotated (follow‐up scan). A double examination was taken while the patient had their leg externally rotated. The CT scanning parameters were set to a tube voltage of 120 kVp, a rotation time 0.5 s, pitch of 0.83, with 80 detector rows at a slice thickness of 0.5 mm and an increment of 0.3 mm, and a matrix size of 512 × 512. The field of view encompassed only the index leg and varied slightly across patients, resulting in an average pixel size of 0.45 mm ± 0.04 mm. The effective dose was estimated using the dose length product (DLP) multiplied by 0.015 mSv/mGy. cm, the k conversion factor of the pelvis.^24^ The estimated mean ± standard deviation of the effective dose of each CT scan was 1.51 ± 0.80 mSv. Scans were processed with single energy metal artifact reduction (SEMAR) and reconstructed using Advanced Intelligent Clear‐IQ Engine (AiCE), a deep‐learning reconstruction technology (Canon Medical Systems Corporation).^25^

**Figure 1:**
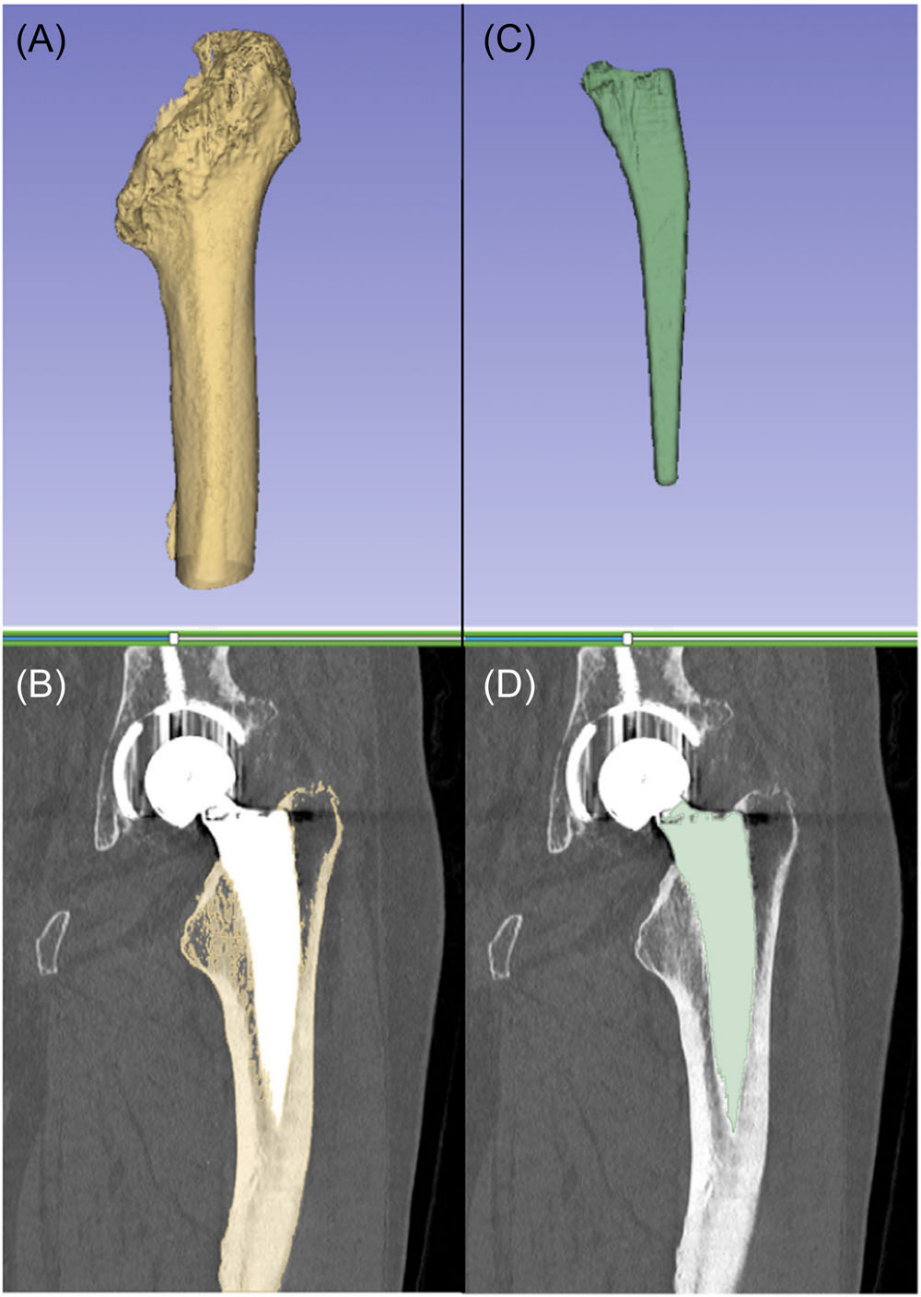
3D slicer results: (A) segmented three‐dimensional model of the femoral bone with (B) coronal in‐plane slice, and (C) segmented three‐dimensional model of femoral stem with (D) coronal in‐plane slice, creating the reference and migrating masks for CT‐RSA. CT‐RSA, computed tomography‐based RSA.

**Figure 2:**
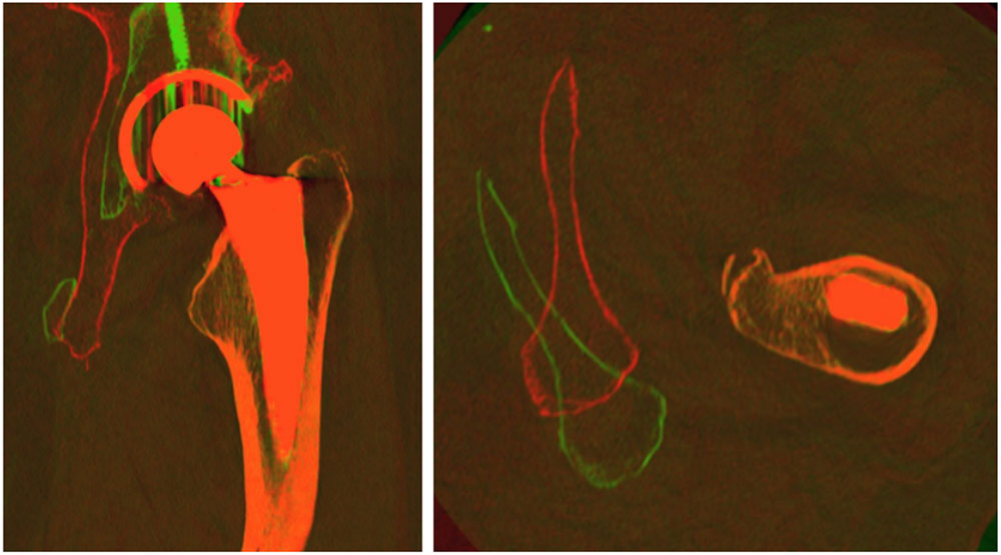
CT‐RSA software with a reference image (external rotation in green) overlaid onto a follow‐up image (internal rotation in red) after registration of migrating object (femoral stem) component mask, in coronal and axial views. CT‐RSA, computed tomography‐based RSA.

The femoral stem and femur bone were segmented from the external rotation CT scan volume using open‐source segmentation software (Slicer. org). The segmentation process began by using a scissors tool to define a 3D region of interest (ROI) around the femoral stem. The stem was subsequently segmented by applying a threshold based on component intensity characteristics within the established ROI. Similarly, a 3D ROI was defined around the femur bone and a threshold was applied to segment the bone within the ROI. Logical operators were used to ensure there was no overlap in segmented components. An example of the resulting segmented component masks is shown (Figure 1). The CT scan volumes and component masks were then loaded into Volumetric Matching Micromotion Analysis (V3MA), a novel CT‐RSA software (RSAcore) used to measure implant movement. V3MA uses image registration to register each component mask from a follow‐up scan to the corresponding component in the reference scan. The result of this registration is exemplified, which shows a reference image overlaid with the follow‐up image after registration of the femoral stem component mask (Figure 2). The CT coordinate system uses a Digital Imaging and Communication in Medicine (DICOM) coordinate system, which is different than the RSA coordinate system. Before analysis, data from left‐sided prostheses was transferred to the right side of the patients for both RSA and CT. Differences between coordinate systems were adjusted by converting the CT coordinate system to the RSA coordinate system. Inducible displacement measurements from CT were calculated in V3MA as the movement of the femoral stem between the alternated external and internal rotation scans.

### 2.4 Data analyses

All statistics were completed using Prism version 10.1.1 (GraphPad Software) and significance was set at a *p*‐value of 0.05 or less. Data normality was assessed using the Shapiro–Wilk test. Where appropriate, either a paired *t*‐test or a Wilcoxon matched‐pair test was used for comparisons. Inducible displacement measurements in each axis of translation and rotation, as well as total translation and total rotation, were compared between RSA and CT‐RSA. To determine if differences in inducible displacement were a result of noise or actual signal, inducible displacement measurements were compared with the double examination measurements for each RSA and CT‐RSA in all axes. Further, measurement error statistics (mean and standard deviation) were calculated from the double examinations for both RSA and CT‐RSA. Since no actual implant migration occurred between the double examinations, the calculated mean represents the measurement bias, and the standard deviation describes the measurement precision.^26^ Precision was calculated and reported as 1.96 times the standard deviation of the double examinations, providing the upper 95% confidence interval (CI).^13^, ^27^ Measurements from the double examination were compared between RSA and CT‐RSA in each axis of translation and rotation, as well as total translation and total rotation.

## 3 RESULTS

**Figure 3:**
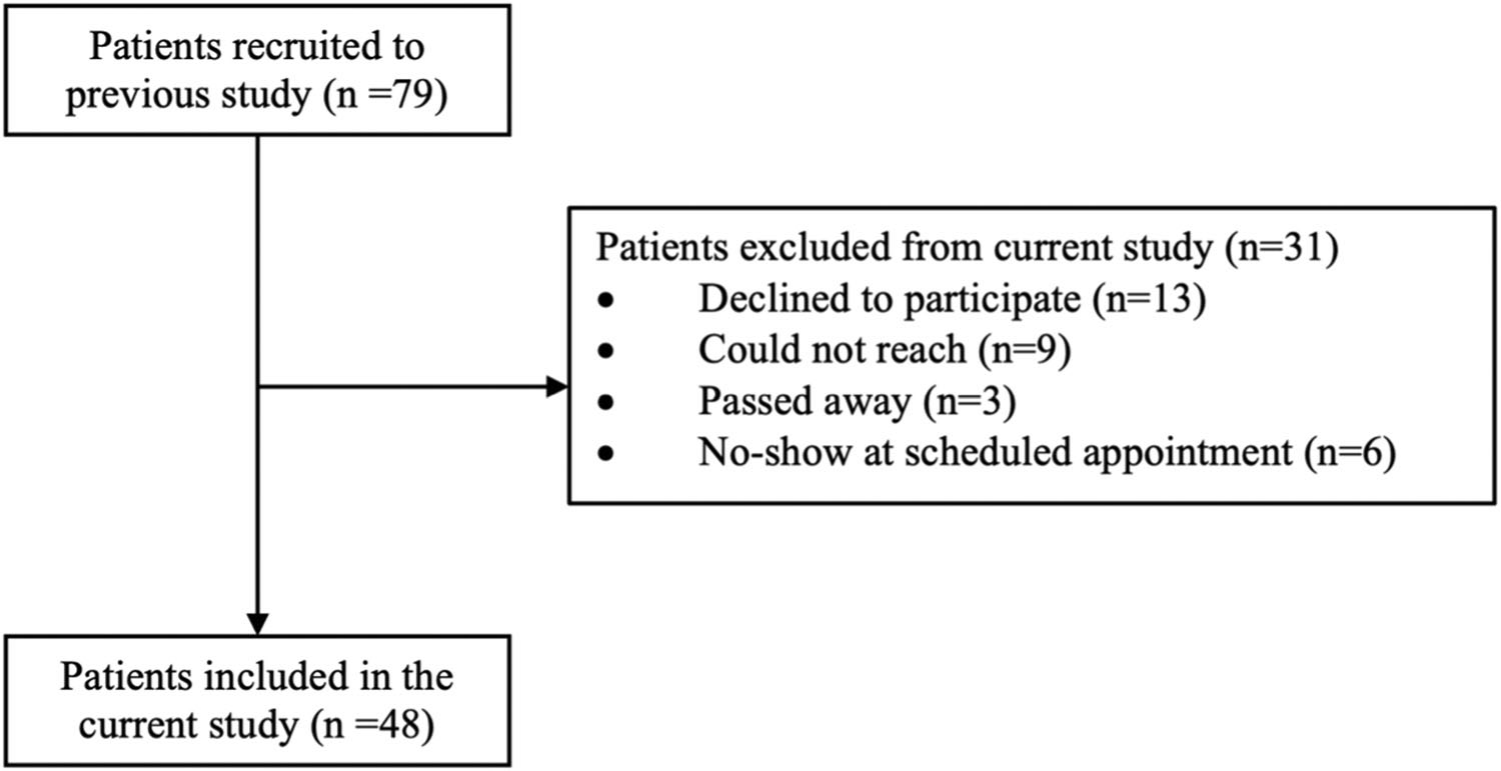
CONSORT study flow diagram.

**Figure 4:**
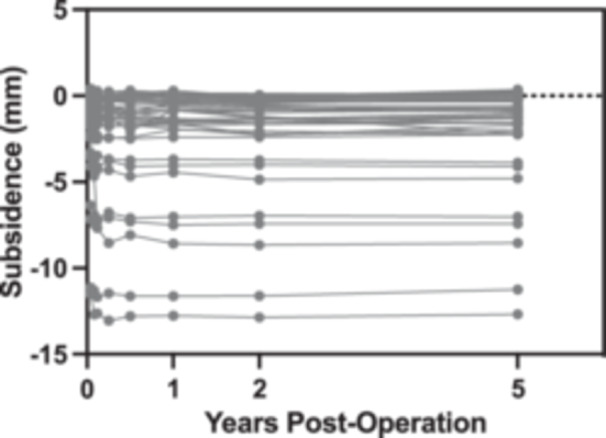
Longitudinal distal (y axis) migration of the femoral stem from the day‐of‐surgery baseline exam to five‐years post‐total hip arthroplasty measured with Model‐Based RSA.

A total of 48 patients provided written informed consent to participate in this 5‐year follow‐up study and underwent both RSA and CT‐RSA imaging (Figure 3). The mean ± standard deviation of femoral stem subsidence between two and 5 years was 0.097 ± 0.197 mm. All patients experienced less than 0.2 mm of subsidence per year between the second and fifth years of follow‐up, indicating they all had well‐fixed, stable implants (Figure 4).

**Figure 5:**
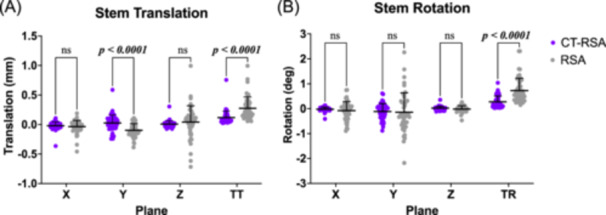
Inducible displacement measurements of the femoral stem measured for CT‐RSA (scans in alternated leg rotations) and RSA (scans in supine and standing). (A) Translation measurements in each axis and total translation (TT). (B) Rotation measurements in each axis and total rotation (TR). Lines and error bars represent mean and standard deviation values, respectively. CT‐RSA, computed tomography‐based RSA; ns, not significant.

There were no differences in inducible displacement measurements in the x or z axes of translation or rotation in any axis between RSA and CT‐RSA, but there were differences for distal (y axis) translation, total translation, and total rotation (Figure 5). Compared to CT‐RSA, RSA demonstrated greater distal translation (mean difference = 0.122 mm, *p* < 0.0001), greater total translation (mean difference = 0.158 mm, *p* < 0.0001), and greater total rotation (mean difference = 0.449°, *p* < 0.0001). There were no differences between the inducible displacement measurements and the double examination of CT‐RSA in any individual axis of translation or rotation, but there was a difference for total translation (mean difference = 0.061 mm, *p* = 0.0003) and total rotation (mean difference = 0.173°, *p* < 0.0001). For RSA, significant differences between inducible displacement and the double examination measurements were found for medial (x axis) translation (mean difference = 0.044 mm, *p* = 0.0042), distal translation (mean difference = 0.104 mm, *p* < 0.0001), total translation (mean difference = 0.132 mm, *p* < 0.0001), and total rotation (mean difference = 0.390°, *p* < 0.0001), suggesting true measured signal.

**Figure 6:**
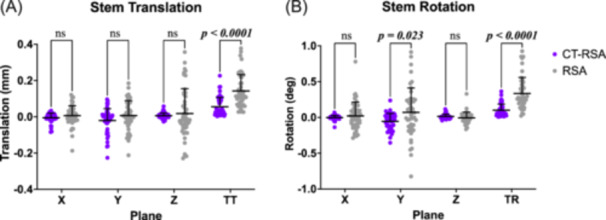
Double examination measurements of the femoral stem for CT‐RSA (scans in external rotation) and RSA (scans in supine). A) Translation measurements in each axis, as well as total translation (TT). B) Rotation measurements in each axis, as well as total rotation (TR). Lines and error bars represent mean and standard deviation values, respectively. CT‐RSA, computed tomography‐based RSA; ns, not significant.

The measurement error statistics of RSA and CT‐RSA are analyzed and reported (Table 2). The precision of RSA ranged from 0.108 to 0.269 mm in translational planes, with a precision of 0.176 mm for total translation. In rotational planes, the precision of RSA ranged from 0.151° to 0.670°, with a precision of 0.441° for total rotation. In comparison, CT‐RSA exhibited superior precision across its double examination in all axes. The precision of CT‐RSA ranged from 0.031 to 0.130 mm in translational planes, with a precision of 0.100 mm for total translation. In rotational planes, the precision of CT‐RSA ranged from 0.061° to 0.220°, with a precision of 0.167° for total rotation. There were no differences in the double examination measurements in any axis of translation or in the x or z axis of rotation between RSA and CT‐RSA, but there were differences for internal (y axis) rotation, total translation, and total rotation (Figure 6). Compared to CT‐RSA, RSA exhibited significantly greater measurement bias for internal rotation (mean difference = 0.124°, *p* = 0.023), total translation (mean difference = 0.087 mm, *p* < 0.0001), and total rotation (mean difference = 0.233°, *p* < 0.0001).

**Table 2:** Measurement error statistics from double examination measurements for RSA and CT‐RSA, where mean indicates the measurement bias and standard deviation describes the measurement precision.

Axis	RSA (*n* = 48)			CT‐RSA (*n* = 48)		
	Mean (Bias)	Standard deviation	Precision (1.96xSD)	Mean (Bias)	Standard deviation	Precision (1.96xSD)
Translation x (mm)	0.007	0.055	0.108	−0.004	0.025	0.049
Translation y (mm)	0.008	0.081	0.159	−0.021	0.066	0.130
Translation z (mm)	0.019	0.137	0.269	0.006	0.016	0.031
Total Translation	0.142	0.090	0.176	0.055	0.051	0.100
Rotation x (°)	0.021	0.191	0.374	−0.002	0.031	0.061
Rotation y (°)	0.069	0.342	0.670	−0.055	0.112	0.220
Rotation z (°)	−0.006	0.077	0.151	0.013	0.032	0.062
Total rotation	0.335	0.225	0.441	0.102	0.085	0.167

Abbreviation: CT‐RSA, computed tomography‐based RSA; RSA, radiostereometric analysis.

## 4 DISCUSSION

With the increased interest in CT‐RSA and a need for a more definitive diagnostic tool for aseptic loosening, the purpose of this study was to evaluate CT‐RSA for inducible displacement measurements of the femoral stem in comparison to the gold‐standard RSA.

The longitudinal migration results revealed that none of the implants exhibited migration greater than 0.2 mm per year, with mean subsidence of 0.097 mm from two‐ to 5‐years postoperation, suggesting all patients had well‐fixed implants.^28^ This is in line with prior longitudinal studies of the Corail cementless femoral stem, such as Critchley et al, who reported a mean subsidence of 0.05 mm (ranging from −0.14 to 0.57 mm) from 6 months to 14 years postoperation.^29^ For these well‐fixed implants, inducible displacement measurements were expected to be minimal in magnitude and attributed to a combination of bone elasticity and measurement error.

This study's findings for inducible displacement found a difference in distal translation along the y‐axis between RSA and CT‐RSA. Measurements from RSA revealed a mean 0.122 mm greater distal displacement of the femoral stem compared to CT‐RSA. Though this magnitude of difference is small, there was also a difference between RSA's inducible displacement and the double examination in this axis, suggesting this difference is actual displacement, and not likely to be measurement error. Further, the difference between RSA and CT‐RSA in this axis may be attributed to the variation in applied external loads, as inducible displacement measurements for RSA were measured between supine and weight‐bearing exams, where displacement is most likely to be distal translation.

There were no other differences in inducible displacement measurements between RSA and CT‐RSA in the other translational or rotational axes. However, RSA measured significantly greater total translation and total rotation than CT‐RSA, likely due to the impact of noise on these measurements. Since total translations and total rotations are unsigned, positive values, the variance of measurements in each axis add positively to the total mean, unlike signed values where variations can offset each other. Consequently, total translations and total rotations increase with the amount of noise, resulting in larger values for RSA than for CT‐RSA, which has superior reported precision. Still, there were differences in total translation and total rotation between inducible displacement measurements and the double examinations for both RSA and CT‐RSA, suggesting true measured signal in the noise.

In recent literature, CT‐RSA has emerged as a prospective alternative to RSA for monitoring implant migration, demonstrating equivalent to superior precision. A study of migration of acetabular cups by Broden et al found comparable precision between a commercially available CT‐RSA software (Sectra AB) and RSA for translations and greater precision with CT‐RSA for rotations.^13^ Likewise, a cadaveric phantom study with a tibial implant by Engseth et al, compared precision between RSA and CT‐RSA and found greater precision with CT than with RSA.^12^ Our results agree with these studies, as we also found that the measurement precision of CT‐RSA was superior to RSA and ranged from 0.031 to 0.130 mm in translational planes and 0.061° to 0.220° in rotational planes. Moreover, a review paper by Kärrholm indicated that RSA precision to the 99% significance interval in clinical studies varied between 0.15 and 0.60 mm for translations and 0.3° to 2° for rotation.^30^ The precision of RSA in our study ranged from of 0.108 to 0.269 mm in translation and 0.151° to 0.670° in rotation, aligning with the reported ranges in Kärrholm's review paper. Further, the error statistics of total translation and total rotation are informative parameters for evaluating and contrasting measurement errors, as outlined by Niesen et al.^26^ In our study, CT‐RSA demonstrated lower bias and superior precision for both total translation and total rotation than RSA, suggesting greater reliability and less noise in the measured data.

Use of CT‐RSA with inducible displacement techniques is a promising tool for analyzing suspected aseptic loosening of orthopaedic implants and can assess both the extent and the characteristics of loosening. However, thresholds of displacement that would indicate loosening of implant components have yet to be established. A previous study of the tibial component with RSA found inducible displacements of up to 1.7 mm of maximum total point motion in well‐fixed knees, perhaps suggesting an upper threshold of displacement acceptance.^31^ Olivecrona et al estimated movement of their diagnosed and revised loose acetabular cups was between 1 mm and 3 mm.^32^ Sandberg et al used their experience to suggest a threshold of 0.5 mm of displacement being detectable with CT‐RSA technology, with smaller displacements down to 0.3 mm being detectable but more challenging to interpret.^33^ In the present study, inducible displacement measurements utilizing CT‐RSA yielded a mean total translation of 0.116 mm and a mean total rotation of 0.275°, yet all these implants were considered well‐fixed. Consequently, this study offers initial data to help establish thresholds for calling a femoral stem loose based on inducible displacement measurements obtained through CT‐RSA examinations.

Some limitations can be noted in the presented study. Namely, since all patients had well‐fixed implants, this study lacked instances to assess the reliability and validity of CT‐RSA in detecting aseptic loosening, limiting the generalizability to cases with confirmed implant stability. Still, findings of our study indicate that the precision of CT‐RSA allows for the detection past the threshold for clinically significant micromotion and provides results for well‐fixed stems that will help to establish the CT‐RSA diagnostic threshold for femoral stem aseptic loosening. Additionally, the comparisons of inducible displacement between RSA and CT‐RSA differ due to the supine and weight‐bearing position for RSA, and the supine position with alternated leg rotations for CT‐RSA. While not directly comparable, both offer meaningful representations of inducible displacement and align with the current clinically relevant methods for assessing inducible displacement in THA with these modalities. Future inducible displacement assessments of the hip with CT while standing may be possible with the recent advancements in weight‐bearing CT scanners that have the capability of scanning the pelvis region.^34^, ^35^ Additionally, future studies should consider incorporating RSA imaging in a supine position with alternated leg rotations to provide a more direct comparison to CT‐based RSA loading protocols. Further, the force of inducible displacement from standing or from alternated leg rotations can be inconsistent and likely varies between patients, suggesting that it might be beneficial to apply a controlled load in future studies. Lastly, the estimated mean effective dose of each CT scan in this study was 1.51 mSv, higher than the recommended sub‐1 mSv low dose that has been previously used in CT‐RSA hip studies.^27^ This difference in radiation dosage highlights the need for careful consideration when designing the CT protocol, especially for examination of the hip. Future work should investigate the performance of the CT‐RSA software at lower doses and with other implant materials, considering potential impact on image quality and metal artifacts.

In conclusion, this study is one of the first to assess the use and precision of CT‐RSA in comparison to the gold‐standard, RSA, for inducible displacement measurements of the femoral stem. Measurements of rotationally loaded inducible displacement from CT‐RSA were minimal, consistent with a cohort of patients with stable, well‐fixed implants. Additionally, CT‐RSA demonstrated superior precision compared to RSA. Although findings of this study are encouraging, additional research that includes patients with loose implants who are candidates for revision surgery is essential to confirm the reliability of CT‐RSA as a diagnostic method for detecting suspected aseptic loosening.

## AUTHOR CONTRIBUTIONS

Jennifer S. Polus, Bart L. Kaptein, Matthew G. Teeter are the conception and design of the study, or acquisition, analysis, and interpretation of data. All authors have drafting or revising the article critically for important intellectual content. All authors have the final approval of the version to be submitted.
